# Treatment Strategies for Pilonidal Sinus Disease in Switzerland and Austria

**DOI:** 10.3390/medicina56070341

**Published:** 2020-07-09

**Authors:** Tenzin Lamdark, Raphael Nicolas Vuille-dit-Bille, Isabella Naomi Bielicki, Laura C. Guglielmetti, Rashikh A Choudhury, Nora Peters, Dietrich Doll, Markus M Luedi, Michel Adamina

**Affiliations:** 1 Department of Surgery, Clinic of Visceral and Thoracic Surgery, Cantonal Hospital Winterthur, 8400 Winterthur, Zurich, Switzerland; tenzin.lamdark@ksw.ch (T.L.); laurachiara@me.com (L.C.G.); michel.adamina@ksw.ch (M.A.); 2 Department of Pediatric Surgery, University Children’s Hospital of Basel, 4056 Basel, Switzerland; Isabella.Bielicki@ukbb.ch; 3 Department of Surgery, University of Colorado Hospital, Aurora, CO 80045, USA; rashikh.choudhury@cuanschutz.edu; 4 Faculty of Medicine, Philipps- University of Marburg, 35037 Marburg, Germany; nora3192@gmail.com; 5 Vechtaer Institut für Forschungsförderung VIFF, 49377 Vechta, Germany; markus.luedi@gmail.com; 6 Department of Procto-Surgery, St. Marien-Krankenhaus Vechta, Teaching Hospital of the Hannover University, 49377 Vechta, Germany; doctors@gmx.ch; 7 Department of Anaesthesiology and Pain Medicine, Inselspital, Bern University Hospital, University of Bern, 3010 Bern, Switzerland; 8 Faculty of Medicine, University of Basel, 4051 Basel, Switzerland

**Keywords:** pilonidal disease, survey, Austria, Switzerland

## Abstract

*Background and objective*: No current nationwide consensus exists on pilonidal disease (PD) treatment in Switzerland and Austria. The objective of this study was to assess and compare the spectrum of PD treatment strategies in Switzerland and Austria. *Materials and Methods*: A survey including 196 certified institutions (Switzerland, *N* = 99 and Austria, *N* = 97) was performed. Treatment strategies for both chronic and acute pilonidal disease were investigated, as well as evolution of treatment over the last 20 years. *Results*: In total, 92 of 196 (47%) hospitals participated in the survey. Recurrence rate (20%) was similar between the two countries. In acute pilonidal disease, a two-stage approach with incision and drainage as the first step was preferred over a one-stage procedure in both countries. In Austria, all patients with chronic pilonidal disease were treated as inpatients, whereas 28% of patients in Switzerland were treated on an outpatient basis (*p* = 0.0019). Median length of hospital stay was double in Austria (four days) compared to Switzerland (two days; *p* < 0.001). Primary resection and off-midline closure (*p* = 0.017) and the use of tissue flaps (*p* = 0.023) were performed more commonly in Austria than in Switzerland. Minimally invasive techniques were performed more often in Switzerland than in Austria (52% vs. 4%, *p* < 0.001). Overall, wide excision with secondary wound healing or midline closures declined over the last 20 years. *Conclusion*: Treatment strategies for chronic PD differ between Austria and Switzerland with more and longer inpatient care in Austria, increasingly minimally invasive approaches in Switzerland, and outdated procedures still being performed in both countries. Overall, heterogeneity of practice dominates in both countries.

## 1. Introduction

Pilonidal disease (PD) is a common disease with an estimated incidence of 26 per 100,000 individuals, mostly affecting young men [1]. It was initially hypothesized that PD arises from ingrowing hair forming sinus tracts [2]. More recently, inter-gluteal hairs were found to be stiffer in patients with PD, possibly enabling hair injections into healthy skin and sinus generation [3]. PD may present as a chronic discharging sinus, an acute abscess formation, or as asymptomatic pits [4,5]. Several different treatment options have been proposed over the last several decades for chronic disease including: limited or wide excision [6,7] with consecutive direct closure, and secondary wound healing or tissue flaps [8,9]. Other treatments include injection of fibrin glue [10,11] or phenol [4,12] into sinus tracts. Video-assisted ablation of pilonidal sinus (VAAPS) was described in 2014 where sinus cavity ablation and cleaning are minimally invasively performed under videoscopic guidance [13,14,15]. Acute abscess formation is typically treated by incision and drainage. Whereas some authors routinely perform secondary pilonidal excision after abscess drainage, others think that abscess incision may definitely cure the disease [4,5]. Despite the high incidence of pilonidal disease, consensus on optimal treatment is lacking within the literature [4,5].

The objective of this study was to assess the treatment strategies of pilonidal disease in two European countries and whether a treatment consensus can be identified in daily practice.

## 2. Materials and Methods

### 2.1. Participants

A total of 196 hospitals treating patients suffering from PD were identified: 99 institutions in Switzerland according to the registry of certified training institutions for Surgery of the Swiss Institute for Postgraduate and Further Education in Medicine (SIWF; https://www.fmh.ch/bildung-siwf.html) were identified; and 97 institutions were identified in Austria according to the list of hospitals provided by the Austrian Federal Ministry of Health and Women (Federal Ministry of Health and Women, https://www.sozialministerium.at/site/).

### 2.2. Questionnaire

A multicenter binational survey in Switzerland and Austria was conducted using an online platform (soscisurvey.de, SoSci Survey GmbH, Munich, Germany). The survey was sent to and answered by the head of the surgical department (or their representative) of the queried hospital. Questions covered general information about the hospital, the treatment unit for PD, treatment strategies for acute and chronic pilonidal disease, and the evolution of this treatment (temporal trends) (see Supplementary Material). The survey was conducted between June and July 2016. Hospitals were contacted via email and a link to the survey was provided. A secondary (reminder) email was sent to the non-responders after one month.

### 2.3. Statistics

GraphPad Prism (www.graphpad.com) and GraphPad homepage (https://www.graphpad.com/quickcalcs/contingency1/), as well as SPSS version 25 (IBM corp., Armonk, NY, USA) were used for data analysis.

Continuous variables are reported as mean and standard deviation (SD) or median and interquartile range and compared between the two groups using two-sample independent *t*-tests or Mann–Whitney U test (non-normal data). Normality was assessed using graphical methods (Q–Q plots and histograms) and the Shapiro–Wilk test.

Categorical variables are summarized as frequencies (%) and were compared using Pearson’s chi-squared test or Fisher’s exact test where applicable. *p*-values < 0.05 (two- tailed) were considered statistically significant.

Possible survey responses on treatments of chronic pilonidal disease were based on a 5-point Likert scale assessing the frequency of use of each treatment: 1: always; 2: often; 3: sometimes; 4: seldom; and 5: never, and compared between groups using a two-tailed Fisher’s exact test. *p*-values less than 0.05 were considered statistically significant.

## 3. Results

### 3.1. Response Rate, Size, and Case Load of Participating Hospitals

In total, 92 of 196 (47%) hospitals participated in the survey. Of this cohort, 40 hospitals (43%) were located in Austria and 52 (57%) in Switzerland. Participating hospitals had a median number of 186 beds (ranging from 30 to 1500 beds) with significantly fewer (median 130) beds in Switzerland compared to Austria (median = 263, *p* = 0.001). In total, a median number of 40 surgeries per year were executed for pilonidal disease in the included hospitals (ranging from 5 to 500 surgeries per year). Median number of surgeries in Switzerland (*n* = 30) was not statistically significantly different from Austria (*n* = 50; *p* = 0.056). The mean percentage of relapse surgeries for PD (i.e., number of relapse surgeries divided by primary surgeries) was 18.2 (SD 9.1) in Austria and 19.7 (SD 8.8) in Switzerland, which was comparable between the two countries. The overall percentage of relapse surgeries was 19% (SD 8.9; Table 1).

### 3.2. Treatment of Chronic Pilonidal Disease

Wide excision with secondary wound healing as primary treatment for chronic PD was seldomly or never performed in 45% and 32% of hospitals in Switzerland and Austria, respectively, whereas 36% (Switzerland) and 40% (Austria) of hospitals always or often let the wound heal by secondary intention. Primary excision with midline closure was performed even less frequently in both countries: 69% in Switzerland and 55% in Austria seldom or never closed the wound in the midline after excision. Primary off-midline closure tended to be less frequently performed in Switzerland versus in Austria. Only 5% of hospitals always or often used tissue flaps in Switzerland, whereas tissue flaps were always or often used in Austria in 27% of patients (*p* = 0.023). The Limberg flap was by far the most common type of flap performed both in Switzerland (67%) and in Austria (77%)., Minimally invasive techniques seemed to be more commonly applied in Switzerland than in Austria. When queried about technical details with regard to minimal invasive procedures, limited fistulectomy was most frequently (82%) endorsed in Switzerland, whereas pit-picking was the most frequently (75%) performed procedure in Austria.

None of the included hospitals in Switzerland or Austria used alternative treatment methods such as instillation of Fibrin or Phenol into sinus tracts. Only 2.1% of Swiss and 6.7% of Austrian hospitals used laser coagulation of the sinus tracts as alternative treatment methods. Of Swiss and Austrian hospitals, 19% and 33.3% applied negative pressure therapy to promote wound healing after excision of chronic PD, respectively. Most participants (90.2% in Switzerland and 85.7% in Austria) marked the sinus tracts (using methylene or toluidine blue), whereas less than half (34.1% in Switzerland and 42.9% in Austria) used perioperative antibiotics. The median length of hospital stay was significantly shorter (two days) in Switzerland compared to Austria (four days; *p* < 0.001). Only 28% of hospitals treated PD as outpatients in Switzerland, whereas all included hospitals treated PD as inpatients in Austria (*p* = 0.0019; Table 2).

### 3.3. Operative Technique for Acute Pilonidal Disease

Only a minority of participating hospitals (13.3% in Switzerland and 23.3% in Austria) used complete excision with an intended one-stage procedure to treat acute PD. Similarly, only 20% of participants (20% in both Switzerland and Austria) used incision and drainage without consecutive secondary excision to treat acute PD. Most participating hospitals (64% in Switzerland and 67% in Austria) applied a two-stage procedure (with incision and drainage of the acute abscess formation and secondary excision) for acute PD. The exact procedure during secondary excision (i.e., excision and primary closure vs. excision and healing by secondary intention vs. excision and flap reconstruction) was not assessed in the present survey (Table 3).

### 3.4. Chronic PD Treatment 20 Years Ago vs. Now

Currently, wide excision with secondary wound healing is performed less frequently both in Switzerland and in Austria than 20 years ago. Similarly, primary midline closures have declined over time in Switzerland and Austria. For off-midline closures, the trend runs in the opposite direction with Swiss surgeons performing less off-midline closures in favor of minimally invasive techniques, and Austrian surgeons performing off-midline closures more frequently than two decades ago. Lastly, use of tissue flaps has increased over time, with now almost every second Swiss surgeon and more than half the Austrian surgeons taking advantage of tissue flaps (Table 4).

## 4. Discussion

In the present study, a total of 92 hospitals representative of Swiss (*n* = 52) and Austrian (*n* = 40) practice reported their treatment strategies for acute and chronic PD. The median number of primary procedures (40 yearly) per hospital and rate of surgery for recurrent PD (about 20%) were similar between the two countries and reflected the real-life data outside of dedicated expert centers. In acute PD, there was consensus among Swiss and Austrian surgeons in performing a two-step approach with incision as a first step, in accordance with current evidence [16,17].

In chronic PD, surgeons in both countries generally performed wide excision with secondary wound healing in about one-third of cases. Both Swiss and Austrian surgeons preferred off-midline closure techniques to midline sutured closures. This is in accordance with the current evidence that off-midline closure techniques are associated with lower complications rates, healing time, and recurrence rates [18,19,20,21]. Notably, in Austria, primary resection and off-midline closure and the use of tissue flaps were performed significantly more frequently compared to Switzerland. In both countries, the Limberg flap was the preferred technique if flap treatment was required. In contrast to Austria, Swiss surgeons largely preferred conducting minimally invasive approaches in chronic PD, with fistulectomy being the most frequently performed technique in Switzerland and pit picking being the preferred technique in Austria. Compared with PD treatment in former times, surgeons in contemporary practice less frequently performed wide excision with secondary wound healing and midline closures.

The present survey was answered by the head of the surgical department or their representative. Although the views of the departmental head may not always be followed by all staff surgeons and residents, compliance with departmental treatment policy is generally very high in the Swiss and Austrian health care systems. Most continental European hospitals comply with a uniform treatment doctrine, as opposed to the U.K. and North American consultant-based health care system, where heterogeneity of practice within a given institution is common. Similarly, Swiss and Austrian hospitals are obliged to yearly report the number of procedures performed for a given disease, providing accurate estimates of caseload.

Regarding the overall response rate, about every second hospital provided its response. Representativeness of the results can hence be assumed. The observed response rate of 47% is higher than the typical response rate of 30–40%, which is expected for an external survey well targeted to its population of interest [22,23,24]. A survey sent to >60,000 Canadian physicians showed an overall response rate of 32% [25], whereas a meta-analysis summarizing the response rates to surveys among surgery residents in the U.S. reported an average response rate of 43% among nationwide surveys [26]. Response rates to surveys differ among disciplines, being even lower than 30% among general surgeons, possibly reflecting their busy daily schedule [22,27]. Nevertheless, the present survey’s results may be confounded by an unequal nonresponse among participants, leading to sampling bias. For example, more experienced surgeons in the field of PD might have a lower nonresponse rate and may be more prone to try innovative therapeutic approaches (e.g., limited fistulectomy or laser assisted procedure).

Both Switzerland and Austria are located in the center of Europe and are dominated geographically by the Alps, share a common culture, and have a similar life expectancy (Austria 81.3 years, Switzerland 83.0 years). Although both countries have a nearly identical population size (AT 8.7 million vs. CH 8.5 million), Switzerland is much more densely populated than Austria (216 vs. 106 per km^2^), with 87% of the Swiss population living in urban centers compared to only 58% in Austria. The Austrian health system provides universal coverage, with low out-of-pocket spending. In contrast, in Switzerland out-of-pocket payments constitute 5.3% of final household consumption, which is among the highest share in the Organization for Economic Co-operation and Development (OECD).

The total number of hospitals in Switzerland and Austria are comparable (283 vs. 273). Nonetheless, Austria has a significantly higher number of hospital beds than Switzerland (7.55 vs. 4.58 per 1000), whereas outpatient consultations and ambulatory surgery are below OECD averages in Austria. The Austrian health system’s focus on inpatient care is also reflected in the number of surgeons per patient, which is significantly higher in Austria than in Switzerland (1 vs. 0.8 per 1000).

In this context, PD was uniformly approached as an inpatient treatment in Austria and that length of hospital stay was double compared to Switzerland, where about one-third of PD patient are treated in ambulatory care. Minimal invasive treatment of chronic PD can be performed in local anesthesia and is generally performed on an outpatient basis. As outpatient treatment of PD is almost never performed in Austria, it is not surprising that these techniques were less frequently performed. Minimally invasive surgery in PD is less invasive and an earlier return to work is possible than with excisional techniques [28]. Minimally invasive surgery has a higher recurrence rate compared to excisional surgery [17,19]. Thus, it is primarily recommended for small lesions that have not been surgically treated before [17]. As there are limited data comparing the different minimally invasive techniques in chronic PD, the choice is left to the surgeons’ preference. The observed preference for fistulectomy in Switzerland may be associated with it first being described by Swiss surgeons [7]. Another minimally invasive technique using video-assisted ablation of PD was described in 2014 in Italy showing promising results [13,14,15]. Nevertheless, VAAPS was not assessed in the present survey.

## 5. Conclusions

This nationwide survey showed that PD caseload and recurrence rates are comparable between Switzerland and Austria. Outdated procedures, such as midline closures and open wound treatments after primary resection, are still performed by some surgeons in both countries, but less commonly than 20 years ago. As there is no consensus in the current published literature on the choice of therapy for chronic PD; the preferred technique likely depends on the surrounding socio-economic and demographic conditions of a treatment center. As outpatient treatment is trending in Switzerland, minimally invasive techniques are more often performed. In Austria, more radical surgery with excision and off-midline closure or flap-treatment, which generally requires inpatient treatment and may have a lower recurrence rate, are preferred over minimal invasive techniques. The percentages of surgery for recurrent PD were similar between Austria and Switzerland. Overall, a dominant or consensus treatment strategy for PD remains elusive, which is reflected in the heterogeneity of treatment modalities reported in the present study.

## Figures and Tables

**Table 1:** Hospital number, size, and surgical volume.

	Switzerland *N* = 52	Austria *N* = 40	All *N* = 92	*p*-Value
Number of hospital beds (median, IQR)	130 (95–300)	263 (170–500)	186 (108–350)	0.001
Number of primary PD surgeries/year (median, IQR))	30 (20–50)	50 (30–70)	40 (25–60)	0.056
Number of relapse surgeries/year (median, IQR))	6 (3–10)	6 (5–10)	6 (5–10)	0.536
Percentage of surgeries for recurrent PD/year (mean, SD)	19.7 (8.8)	18.2 (9.1)	19 (8.9)	0.500

IQR = interquartile range, PD = pilonidal disease, SD = standard deviation.

**Table 2:** Treatment of chronic pilonidal disease.

	Switzerland	Austria	All	*p*-Value
Secondary wound healing, *n* (%)	47 (100%)	28 (100%)	75 (100%)	
• Always	3 (6%)	3 (11%)	6 (8%)	0.665
• Often	14 (30%)	8 (29%)	22 (29%)	1
• Sometimes	9 (19%)	8 (29%)	17 (23%)	0.399
• Seldom	14 (30%)	7 (25%)	21 (28%)	0.792
• Never	7 (15%)	2 (7%)	9 (12%)	0.47
Midline wound closure, *n* (%)	45 (100%)	29 (100%)	74 (100%)	
• Always	0 (0%)	2 (7%)	2 (3%)	0.15
• Often	1 (2%)	2 (7%)	3 (4%)	0.557
• Sometimes	13 (29%)	9 (31%)	22 (30%)	1
• Seldom	12 (27%)	4 (14%)	16 (22%)	0.252
• Never	19 (42%)	12 (41%)	31 (42%)	1
Off-midline wound closure, *n* (%)	45 (100%)	28 (100%)	73 (100%)	
• Always	0 (0%)	3 (11%)	3 (4%)	0.053
• Often	5 (11%)	7 (25%)	12 (16%)	0.193
• Sometimes	6 (13%)	7 (25%)	13 (18%)	0.225
• Seldom	17 (38%)	3 (11%)	20 (27%)	0.015
• Never	17 (38%)	8 (29%)	25 (34%)	0.458
Wound closure with tissue flaps, *n* (%)	40 (100%)	26 (100%)	66 (100%)	
• Always	0 (0%)	5 (19%)	5 (8%)	0.0074
• Often	2 (5%)	2 (8%)	4 (6%)	0.644
• Sometimes	12 (30%)	5 (19%)	17 (26%)	0.397
• Seldom	16 (40%)	8 (31%)	24 (36%)	0.601
• Never	10 (25%)	6 (23%)	16 (24%)	1
Type of flap, *n* (%)				
• Limberg flap	24 (66.7)	17 (77.3)	41 (70.7)	0.554
• Modified Limberg flap	8 (22.2)	2 (9.1)	10 (17.2)	0.29
• Other	4 (11.1)	3 (13.6)	7 (12.1)	1
Minimal invasive treatment, *n* (%)	42 (100%)	13 (100%)	55 (100%)	
• Always	9 (21%)	0 (0%)	9 (16%)	0.096
• Often	13 (31%)	1 (8%)	14 (25%)	0.147
• Sometimes	9 (21%)	3 (23%)	12 (22%)	1
• Seldom	6 (14%)	8 (62%)	14 (25%)	0.0017
• Never	5 (12%)	1 (8%)	6 (11%)	1
Type of minimal invasive treatment, *n* (%)	31 (81.6)			
• Fistulectomy	4 (10.5)	2 (16.7)	33 (66)	<0.001
• Pit picking	3 (7.9)	9 (75)	13 (26)	<0.001
• Other		1 (8.3)	4 (8)	1
Instillation of Fibrin or Phenol into sinus tracts, *n* (%)	0	0	0	-
Laser instillation of sinus tracts, *n* (%)	1 (2.1)	2 (6.7)	3 (3.9)	0.557
Negative pressure therapy, *n* (%)	9 (19)	10 (33.3)	19 (24.7)	0.183
Marking of sinus tracts (methylene or toluidine blue), *n* (%)	37 (90.2)	24 (85.7)	61 (88.4)	0.706
Use of perioperative antibiotics, *n* (%)	14 (34.1)	12 (42.9)	26 (37.7)	0.613
Length of hospital stay, median (IQR), (days)	2 (1–3)	4 (2–4)	3 (2–4)	< 0.001
Percentage of same-day surgeries	28%	0%	16%	0.0019

**Table 3:** Treatment of acute pilonidal disease (PD).

	Switzerland	Austria	All	*p*-Value
Primary complete excision of acute PD, *n* (%)	6 (13.3)	7 (23.3)	13 (17.3)	0.353
Incision only for acute PD, *n* (%)	9 (20)	6 (20)	15 (20)	1.000
Two-stage procedure for acute PD, *n* (%)	29 (64.4)	20 (66.7)	49 (65.3)	1.000

**Table 4:** Chronic pilonidal disease treatment 20 years ago versus now.

	Switzerland	Austria	All	*p*-Value
Wide excision with secondary wound healing, *n* (%)				
- more often	3 (8)	2 (8)	5 (7)	1
- same frequency	11 (27)	6 (23)	17 (26)	0.778
- less common	26 (65)	18 (69)	44 (67)	1
Midline wound closures, *n* (%)				
- more often	7 (18)	6 (24)	13 (20)	0.541
- same frequency	8 (20)	4 (16)	12 (18)	0.751
- less common	25 (62)	15 (60)	40 (62)	1
Off-midline wound closures, *n* (%)				
- more often	10 (26)	14 (52)	24 (36)	0.039
- same frequency	5 (13)	2 (7)	7 (11)	0.691
- less common	24 (61)	11 (41)	35 (53)	0.133
Flap repair, *n* (%)				
- more often	19 (48)	15 (58)	34 (52)	0.455
- same frequency	7 (17)	5 (19)	12 (18)	1
- less common	14 (35)	6 (23)	20 (30)	0.291

## Supplementary Materials

The following questionnaire is available online: https://www.mdpi.com/1010-660X/56/7/341/s1. Questionnaire to assess Treatment Strategies for Pilonidal Sinus Disease in Switzerland and Austria.
