# Supplementary material for: Treatment Strategies for Pilonidal Sinus Disease in Switzerland and Austria
Source: Medicina (Kaunas). 2020 Jul 9;56(7):341. doi: 10.3390/medicina56070341 (PMC7404453; doi:10.3390/medicina56070341)
Supplement: Supplementary file 1 [file medicina-56-00341-s001.pdf]

Questionnaire to assess Treatment Strategies for Pilonidal Sinus Disease in Switzerland and Austria

1. How many beds has Your hospital?

2. Are patients with pilonidal Sinus being operated in Your hospital?

○ Yes

○ No (If no, please terminate the questionnaire now.)

3. Which discipline is treating pilonidal disease at your institution?

☐ Visceral Surgery

☐ General Surgery

☐ Dermatology

☐ Plastic Surgery

☐ Other:

4. Which FMH category has this department?
Question only for Switzerland.

○ U

○ A

○ B

○ C

5. How many beds are available in the department treating pilonidal disease?

6. How many operations for pilonidal disease are conducted by this department per year?

7. How many of these operations are performed due to recurrence?

8. Who is mainly treating patients with pilonidal disease during daytime?

(Multiple options are possible)

- ☐ Head of department / Consultant Surgeon
- ☐ Attending Surgeon
- ☐ Specialist
- ☐ Resident
- ☐ External Surgeon
- ☐ ______

9. Who is mainly treating patients with pilonidal disease on duty?

(Multiple options are possible)

- ☐ Head of department / Consultant Surgeon
- ☐ Attending Surgeon
- ☐ Specialist
- ☐ Resident
- ☐ External Surgeon
- ☐ ______

10. What is Your own position?

(Multiple options are possible)

- ☐ Head of department / Consultant Surgeon
- ☐ Attending Surgeon
- ☐ Specialist
- ☐ Resident
- ☐ External Surgeon
- ☐ ______

Treatment of chronic pilonidal disease

Please mark on the scale below, if and how often your department uses the following therapies for chronic pilonidal disease:

11. Primary open technique (Wide excision and secondary wound healing)

(Multiple options are possible)

○ never ○ rarely ○ sometimes ○ often ○ always

12. If You use the primary open technique, which one is it?

(Multiple options are possible)

☐ Excision

☐ Excision and marsupialisation (stitching down) of the wound rim

☐ Others ______

13. What arguments favor the primary open technique in your opinion?

(Multiple options are possible)

○ short operating time

○ simple execution

○ low costs

○ short hospital stay

○ low recurrence rate

○ ______

14. In your opinion, what speaks against the primary open technique?

(Multiple options are possible)

- ○ long treatment time
- ○ high treatment costs
- ○ pain by more scar tissue
- ○ ____________

15. Primary midline closure

○	○	○	○	○
never	rarely	sometimes	often	always

16. Primary off-midline closure

○	○	○	○	○
never	rarely	sometimes	often	always

17. If primary closure is performed, which technique is used?

(Multiple options are possible)

- ○ Karydakis
- ○ Bascom
- ○ Other: ____________

18. What arguments favor the primary closure technique in your opinion?

(Multiple options are possible)

- ○ short hospital stay
- ○ re-integration into the (working) life
- ○ reduced formation of scar tissue
- ○ shorter treatment duration
- ○ lower treatment costs
- ○ ____________

18. In your opinion, what speaks against primary wound closure?

(Multiple options are possible)

- ◯ higher recurrence rate in paramedial primary closure
- ◯ higher recurrence rate in midline primary closure
- ◯ ____________

20. Use of flaps

◯	◯	◯	◯	◯
never	rarely	sometimes	often	always

21. When you use flaps, which technique is used

(Multiple options are possible)

- ◯ Limberg-flap surgery
- ◯ modified Limberg flap surgery
- ◯ Dufourmentel flap
- ◯ modified Dufourmantel flap

Other:

- ◯ ____________

22. What arguments favor the flap technique in your opinion?

(Multiple options are possible)

- ◯ short hospital stay
- ◯ low recurrence rate
- ◯ low postoperative complication rate
- ◯ faster re-integration into the (working) life
- ◯ other: ____________

23. In your opinion, what speaks against the flap technique?

(Multiple options are possible)

- ◯ long operation time
- ◯ demanding execution
- ◯ high costs
- ◯ long hospital stay
- ◯ high recurrence rate
- ◯ high postoperative complication rate
- ◯ other: __________

24. Use of minimally invasive techniques

(sinusectomy, pit-picking, brushing of the ducts, Trephines and others)

◯	◯	◯	◯	◯
never	rarely	sometimes	often	always

25. If a minimally invasive surgical technique is applied, which one is used?

(Multiple options are possible)

- ◯ Pit-picking
- ◯ Sinusectomy
- ◯ Other: __________

26. What arguments favor minimal invasive techniques in your opinion?

- ◯ short hospital stay
- ◯ faster re-integration into the (working) life
- ◯ reduced formation of scar tissue
- ◯ __________

26. In your opinion, what speaks against the use of minimally invasive techniques?

(Multiple options are possible)

- ○ higher recurrence rate
- ○ longer duration of surgery
- ○ [ ]

28. Are other not previously mentioned techniques used?

- ○ Yes
- ○ No

29. If other, not previously mentioned techniques are applied, which ones are used?

(Multiple options are possible)

- ☐ fibrin instillation
- ☐ phenol instillation
- ☐ laser coagulation oft he sinus ducts
- ☐ excision, vacuum sponge liner
- ☐ other: [ ]

Treatment of acute pilonidal disease

30. Treatment for acute abscess formations in pilonidal disease

(Multiple options are possible)

- ☐ immediate definitive surgery (one-stage intervention)
- ☐ incision and drainage only
- ☐ two stage process: primary incision, secondary elective treatment

  e.g. by ______

31. If you perform an immediate definitive repair (i.e. one-stage treatment), then the following technique is used:

(Multiple options are possible)

- ☐ Excision and primary closure
- ☐ Excision and secondary wound healing
- ☐ Other: ______

Treatment for asymptomatic pilonidal disease

32. Preferred treatment of asymptomatic pilonidal disease

(Multiple options possible)

- ☐ Waiting, for the time being no surgery
- ☐ surgery, namely: ______
- ☐ regular mechanical shaving
- ☐ laser depilation

Treatment adjuncts

33. Do you use antibiotics? Before, during or after surgery?

○ Yes
○ No

34. If an antibiotic therapy is performed, then the following applies…
(Multiple options are possible)

☐ orally
☐ intravenous
☐ local
☐ Single-shot administration
☐ 1-3 days
☐ More than 3 days
☐ Which antibiotic.: ________

35. Do you use of methylene blue / toluidine during surgery?

○ Yes
○ No

36. Is a drain inserted?

○ Yes
○ No

37. Do You insert a resorbable Gentamicin Sponge for a primary closure of the surgical wound or when a flap is performed?

○ Yes
○ No

38. Who is responsible for postoperative care?
(Multiple options are possible)

○ The department / hospital treating the patient
○ An established surgeon
○ The general practitioner

Pilonidal disease treatment 20 years ago versus now

39. Was the surgical procedure different in your hospital 20 years ago?

- ○ If yes. Which techniques were used more frequently in the past? [ ]
- ○ No

40. Is the primary open procedure (wide excision and secondary wound healing) performed more or less often than 20 years ago?

(Please click on the scale)

○	○	○	○	○
less common		just as often		more frequently

41. Is the primary closure in the midline carried out more or less often than 20 years ago?

(Please click on the scale)

○ ○ ○ ○ ○

less common just as often more frequently

42. Is primary off-midline closure performed more or less often than 20 years ago?

(Please click on the scale)

○ ○ ○ ○ ○

less common just as often more frequently

43. Are flaps performed more or less often than 20 years ago?

(Please click on the scale)

○ ○ ○ ○ ○

less common just as often more frequently

Additional questions

44. How long do patients stay in your hospital following pilonidal disease surgery (estimate)?

○ 0 days (outpatient)
○ 1 day
○ 2 days
○ 3 days
○ 4 days
○ 5 days
○ 6 days
○ 7 days
○ 8 days
○ 9 days
○ 10 or more days

45. Do You recommend a depilation by shaving or a laser depilation to prevent recurrence?

☐ Yes
☐ No

46. Which predisposing factors for pilonidal disease exist in your opinion?

(Multiple options are possible)

☐ poor hygiene
☐ obesity
☐ gender
☐ tendency to sweat
☐ family history
☐ steep anal fold
☐ thick, solid hair
☐ recurrent folliculitis
☐ Other: ____________

47. In what canton / federal state is your hospital located?

____

48. Which language region belongs your hospital to?

(Multiple options are possible)

☐ German
☐ French
☐ Italian
☐ Romansh

49. Which legal form does your hospital have?

- ◯ independent official institution (Selbständige öffentliche Anstalt)
- ◯ stock company (Aktiengesellschaft)
- ◯ private foundation (Private Stiftung)
- ◯ dependent public-law institution (unselbständige öffentliche Anstalt)
- ◯ private association (Privater Verein)
- ◯ foundation under public law (Öffentliche Stiftung)
- ◯ special purpose association (Zweckverband)
- ◯ part of the administration (Teil der Verwaltung)
